# Prevalence and risk factors for Hepatitis C and HIV-1 infections among pregnant women in Central Brazil

**DOI:** 10.1186/1471-2334-9-116

**Published:** 2009-07-27

**Authors:** Zelma B Costa, Gustavo C Machado, Mariza M Avelino, Clidenor Gomes Filho, Jose V Macedo Filho, Ana L Minuzzi, Marilia D Turchi, Mariane MA Stefani, Wayner Vieira de Souza, Celina MT Martelli

**Affiliations:** 1 Faculty of Medicine, Federal University of Goiás, Goiás, Brazil; 2 Health State Secretariat, Goiás, Brazil, Associação de Pais e Amigos dos Excepcionais de Goiania – APAE, Goiás, Brazil; 3 Institute of Tropical Medicine and Public Health, Federal University of Goiás, Goiás, Brazil; 4 Oswaldo Cruz Foundation, Centro de Pesquisas Aggeu Magalhães, Pernambuco, Brazil

## Abstract

**Background:**

Hepatitis C (HCV) and human immunodeficiency virus (HIV) infections are a major burden to public health worldwide. Routine antenatal HIV-1 screening to prevent maternal-infant transmission is universally recommended. Our objectives were to evaluate the prevalence of and potential risk factors for HCV and HIV infection among pregnant women who attended prenatal care under the coverage of public health in Central Brazil.

**Methods:**

Screening and counselling for HIV and HCV infections was offered free of charge to all pregnant women attending antenatal clinic (ANC) in the public health system, in Goiania city (~1.1 million inhabitants) during 2004–2005. Initial screening was performed on a dried blood spot collected onto standard filter paper; positive or indeterminate results were confirmed by a second blood sample. HCV infection was defined as a positive or indeterminate sample (EIA test) and confirmed HCV-RNA technique. HIV infection was defined according to standard criteria. Factors associated with HIV and HCV infections were identified with logistic regression. The number needed to screen (NNS) to prevent one case of infant HIV infection was calculated using the Monte Carlo simulation method.

**Results:**

A total of 28,561 pregnant women were screened for HCV and HIV-1 in ANC. Mean maternal age was 23.9 years (SD = 5.6), with 45% of the women experiencing their first pregnancy. Prevalence of HCV infection was 0.15% (95% CI 0.11%–0.20%), and the risk increased with age (p < 0.01). The prevalence of anti-HIV infection was 0.09% (95% CI 0.06%–0.14%). Black women had a 4.9-fold (95% CI 1.42–16.95) greater risk of HIV-1 infection compared to non-black women. NNS to prevent one case of infant HIV infection ranged from 4,141 to 13,928.

**Conclusion:**

The prevalence of HIV and HCV infections were low among pregnant women, with high acceptability rates in the opt-in strategy in primary care. Older maternal age was a risk factor for HCV and antenatal HCV testing does not fulfill the requirements for screening recommendation. The finding of higher risk of HIV-1 infection among black women despite being in consonance with the HIV-1 ethnic pattern in some American regions cannot be ruled out to be a surrogate marker of socio-economic condition.

## Background

Hepatitis C virus and HIV infections are a major burden to public health worldwide and share common blood-borne transmission routes. Approximately 130 million individuals are HCV-infected, with 3.4 million newly infected cases per year, representing a leading cause of liver cancer and transplant worldwide [1]. HIV infection affects around 33 million individuals, with an increasing trend among women that results in mother-to child transmission being the major route for HIV infection in children [2,3]. Among HIV-infected individuals, four to five million are estimated to be co-infected with HCV [1].

Routine antenatal HCV screening to prevent vertical transmission is a controversial issue and is not universally implemented. Although HCV infection is considered an important public health problem for which reliable screening tests exist, treatment is contra-indicated during pregnancy due to the potential risks of the diagnostic procedure [4-7]. In general, antenatal and population screening is recommended for those possessing a risk factor for HCV acquisition, such as intravenous drug users, recipients of blood transfusions or organs before 1990, haemodialysis patients and HIV-infected individuals [8,9]. In Brazil, universal screening is mandatory for blood donors. A few population-based studies have reported anti-HCV prevalence varying from 1.2% to 1.9% [10-13], and the vast majority of studies were restricted to high risk groups in selective areas.

Antenatal HIV screening is universally recommended to prevent vertical transmission [14,15]. The mother-to-child transmission rate varies from 15% to 25% without preventive measures, but this rate can be substantially decreased to 2% or less with the adoption of an evidence-based group of interventions based upon the use of antiretroviral drugs, the avoidance of breastfeeding and elective caesarean section [2,16-18]. However, by 2005, only 11% of HIV-positive pregnant women had gained access to preventive interventions worldwide [19].

After almost three decades of the AIDS epidemic in Brazil, an estimated 620 thousand individuals are living with HIV/AIDS. The prevalence of infection remained stable over the past years, but the increased heterosexual transmission led to a decline of the male-to-female ratio, which reached 1.5 to 1 in 2002 [20,21]. Approximately 90% of the AIDS cases among children (<13 years old) are attributable to mother-to-child-transmission (MTCT). Although the rate of vertical transmission has been declining, the implementation of antenatal HIV screening and timely management of infected pregnant women varies across the country due to disparities of health care access, suggesting missed opportunities for prevention [22-24].

In the current manuscript, we present the prevalence of HCV and HIV infection among pregnant women attending an official program (named Mother's Testing) launched in 2004 that aims to extend the coverage of antenatal screening in public health settings in Central Brazil. This study also analyses potential risk factors for acquisition of these infections associated with maternal characteristics, focusing on the discussion of timely strategies and the estimated outcomes of this screening program.

## Methods

### Study Design, Population and Recruitment

A cross-sectional study was conducted among pregnant women who attended prenatal clinics located in Goiania, a city of ~1.1 million inhabitants in Central Brazil, between 2004 and 2005. Since 2004, screening for nine infectious diseases (including HIV and HCV) has been offered free of charge by an extended Women's Prevention Program sponsored by the State Secretariat of Health (Programa de Protecao a Gestante). This screening program covers all pregnant women who attend the 243 public health centres, which corresponds to 60% of the estimated number of pregnant women in this region. In 1997, the policy of universal screening for HIV and counselling for pregnant women was adopted by the Brazilian Ministry of Health. HCV testing is not part of the routine antenatal screening for vertical transmission prevention.

### Data collection

During the first prenatal visit, a standardized questionnaire is filled in by the health technicians. At the time of blood collection, the following variables are recorded: clinic of attendance; date of maternal birth; last menses; gestational age (weeks); parity; type of previous delivery; number of abortions. Enclosed in the questionnaire was a consent form; informed consent was obtained for HIV-1 and HCV testing as an "opt in" procedure.

### Specimen collection and serological tests

At the first screening, blood samples were collected by digital puncture onto standard filter paper (S&S 903), dried at room temperature (~4 hours) and sent daily by special mail to the Central Reference Laboratory. For confirmatory tests, a second blood venous puncture (vaccutainer) was collected for all pregnant women with positive and indeterminate results in the serological screening using filter paper eluate.

### HCV screening and confirmatory tests

HCV screening was based on the detection of antibodies against HCV core, NS3, NS4 and NS5 antigens (DETECT^® ^commercial kit ADALTIS INC., Canada). As part of the confirmatory procedure the second serum sample obtained from venous puncture was retested by another ELISA kit (Hepanostika HCV Ultra Beijing United Biomedical Co. Ltd., China). In parallel, detection of HCV-RNA was obtained by qualitative AMPLICOR HCV Test (Roche, USA). Samples positive or indeterminate to ELISA and confirmed by HCV-RNA were considered HCV infected.

### HIV screening and confirmatory tests

Serological screening for HIV1/2 in filter paper used an anti-HIV TETRA ELISA commercial kit (Biotest, Germany) to detect anti-HIV1 antibodies (gp41, gp36 and p24) and anti-HIV2 antibodies with sensitivity of 100.0% and specificity of 99.8%, according to the manufacturer. Screened women with positive and indeterminate results were re-tested using sera from venous puncture blood samples (GENSCREEN^® ^HIV1/2 kit Bio-Rad, France) that recognizes antibodies against antigens gp160 and gp25 or by HIV-1/2 ELISA test (Wiener lab., Argentina) that detects antibodies against the pop and gag regions' antigens.

In parallel, at least one of the following confirmatory tests were applied: Indirect Immunofluorecence by IFI – HIV1 kit (Bio-Manguinhos, Fundação Oswaldo Cruz, Rio de Janeiro); Immuno Blot (IB) using a NEW LAV BLOT 1 kit (Bio-Rad, France) that detects antibodies against HIV-1 gp160, gp110/120, p68, p55, p52, gp41, p40, p34, p24/25 and p18; Western Blot (WB) for detection of HIV1 and HIV2, using HIV BLOT 2.2 kit (Genelabs ^® ^Diagnostics Pte Ltd, Singapore) and qualitative "Nested" PCR to detect pro viral HIV1 DNA in blood mononuclear cells (50 copies/mL detection limit), performed at the Center of Genomics Laboratory (São Paulo, Brazil).

A pregnant woman was considered to be HIV-infected if the initial positive and indeterminate screening test was confirmed by anti-HIV1/2 ELISA in a second serum sample combined with a positive result obtained in any other technique (IFI, IB, WB or PCR), as recommended by the Brazilian Ministry of Health [25].

### Data analysis

Data were analyzed by using the chi-square test with Yates' correction for univariate analysis. Prevalence estimates of HCV and HIV infections were reported with 95 percent confidence intervals (95% CI). Outcome measures were HCV and HIV infections. The variables age-group, race/ethnicity, number of pregnancies, mode of delivery and gestational age were evaluated by measuring the strength of association with the outcomes by odds ratios (OR) and their respective 95% CI. Multivariate analysis was conducted by using logistic regression, with anti-HCV and HIV serologic results as the dependent variable (SPSS, Inc., Chicago, Illinois). All variables found to be associated with HCV or HIV infection then were evaluated in a final model, controlling for age.

The Number Needed to Screen (NNS) to prevent one case of mother to child transmission of HIV was calculated using the following parameters: prevalence of HIV infection obtained by the current study; sensitivity of the test (0.99); number receiving test results; number of cases of mother to child transmission expected without interventions among women receiving test results; number receiving combination antiretroviral therapy; number receiving elective cesarean section (C-section) [15,26]. We used the Monte Carlo simulation method to calculate the NNS considering antiretroviral therapy and elective C- section. Briefly, the simulation model uses the above parameters and generates relative risks (RRs) by the combination of preventive measures (combination of antiretroviral regimens and elective cesarean) to avoid vertical transmission. The underlying assumption was the normal distribution of log (RR) and these values transformed back to the original scale were used to estimate NNS and 95% CI based on 1 000 000 samples. The R software was used for the calculations and the written program to perform simulation was kindly provided by Chou et al, 2005.

The large sample size (n = 28,561 pregnant women) of the study is sufficient to produce quite narrow 95% confidence intervals even considering small numbers for prevalence estimates of HCV and HIV infections.

### Ethical issues

Both HIV and HCV tests are offered as part of the routine panel tests in this antenatal screening public health program as an opt-in strategy which require explicit written consent and pretest counseling. The State Public Health Program (Programa de Proteção à Gestante/PPG-Go) ensures the release of test results and medical counselling free of charge and also monitors maternal-child outcomes.

## Results

During the study period, a total of 28,561 pregnant women were screened for HCV and HIV infections in prenatal public health clinics. Only 15 out of 28,576 participants (0.05%) refused to sign the informed consent for HIV and HCV tests. Approximately 85% of the women enrolled were under 30 years of age (mean = 23.9; sd = 5.6), 0.8% were between 12 to 14 years old and approximately 1% were older than 40 years. More than 90% of the study population self-referred as white or mixed/biracial, with 8.3% black and a minority of Asian descendents and indigenous individuals (Table 1).

**Table 1:** Maternal characteristics, prevalence and risk factors for HCV and HIV infection among pregnant women in Public Health Settings, Central Brazil

		HCV		HIV-1	
			
Characteristics	Total N	Positive (%)	OR (95% CI)	Positive (%)	OR (95% CI)
Age, years^a^					
12–19	6664	4 (0.06)	1	2 (0.03)	1
20–29	17084	23 (0.13)	2.2 (0.8 – 6.5)	16 (0.09)	3.1 (0.7 – 13.6)
30–39	4272	9 (0.21)	3.5 (1.1–11.4)	7 (0.16)	5.1 (1.1 – 24.8)
≥ 40	268	4 (1.50)	24.9 (6.2–98.9)		
					
Ethnicity^b^					
White	7710	11 (0.14)	1	5 (0.06)	1
Biracial	9523	20 (0.21)	1.5 (0.7 – 3.3)	10 (0.11)	1.6 (0.6 – 4.7)
Black	1576	1 (0.06)	0.4 (0.02 – 3.3)	6 (0.38)	5.9 (1.8 – 19.2)
Asian/indigenous	176				
					
Number of pregnancies^c^					
First	11637	12 (0.10)	1	9 (0.08)	1
2–3	11079	19 (0.17)	1.7 (0.8 – 3.4)	11 (0.10)	1.3 (0.5 – 3.1)
≥ 4	2780	10 (0.36)	3.5 (1.5 – 8.1)	5 (0.18)	2.3 (0.8 – 6.9)
					
Previous mode of delivery^d^					
Vaginal	7665	14 (0.18)	1	5 (0.07)	1
Cesarean section	4301	13 (0.30)	2.0 (0.9 – 4.6)	7 (0.16)	2.5 (0.7 – 9.0)
Abortions only	1893	2 (0.11)	0.6 (0.1 – 2.6)	4 (0.21)	3.2 (0.9 – 12.0)
					
Gestational age, weeks^e^					
< 14	14647	19 (0.13)	1	11 (0.08)	1
14–27	8741	16 (0.18)	0.8 (0.4 – 1.6)	8 (0.09)	1.3 (0.5 – 3.3)
≥ 28	1077	2 (0.19)	1.1 (0.2 – 4.5)		

Missing data: ^a ^273; ^b ^9576; ^c ^3065; ^d ^3065; ^e ^4096

The maternal characteristics and delivery history are presented in Table 1. Approximately half of the women were experiencing their first pregnancy, and 11% reported four or more pregnancies. For those with a previous history of pregnancies, around 55% reported vaginal delivery and around 30% reported caesarean sections (with or without previous abortions). One third of the women reported having at least one abortion. For the majority of the attendees (95.5%) the first prenatal medical visit, which included the first screening test, occurred before the 27th gestational week and 60% were screened before the 14th week (mean = 13.5; sd = 6.3).

**Figure 1:**
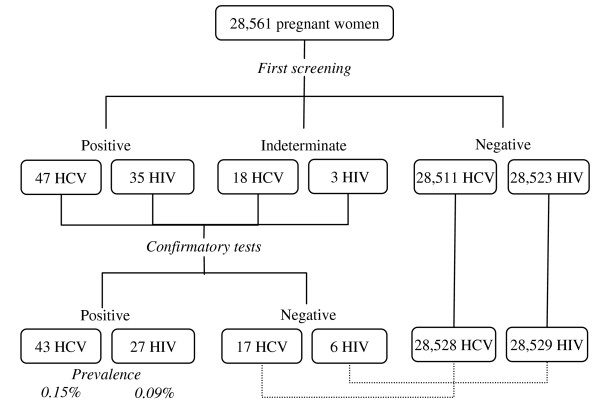
**Screening for HCV and HIV infection among pregnant women in Public Health Settings, Central Brazil**. Five out of 65 anti HCV-positive/indeterminate women and five out of 38 anti-HIV positive/indeterminate women could not be traced for confirmatory test. Doted lines indicate that indeterminate test results (HCV and HIV) were considered negative.

The prevalence of anti-HCV antibodies was 0.22% at the first screening using filter paper eluate. Five out of 65 anti HCV positive or indeterminate women were not re-tested because they changed address and could not be traced. Of the anti-HCV positive women, 92.3% were re-tested for HCV RNA and of these, 72% (43/60) had detectable viremia. The percentage of false-positive results was 28.3% (17/60), and the majority of indeterminate results on the serological screening were not confirmed by molecular test. The prevalence of HCV infection was 0.15% (95% CI 0.11% – 0.20%) at the prenatal public health services (Figure 1).

The prevalence of antibodies to HIV-1 was 0.13% at the initial screening, and 86.8% of the positive participants were re-tested (33/38). Five out of 38 positive or indeterminate women were not re-tested because they changed address and could not be traced. The percent of false positive results was 18.2% (6/33), and all indeterminate results onto filter paper eluate were negative at confirmatory tests. The prevalence of HIV infection was 0.09% (95% CI 0.06%–0.14%) which represents nine infected pregnant women among 10,000 screened (Figure 1). Among HIV-1 positive pregnant women, 34.6% had a history of previous abortion. Two women were HCV-HIV-1 co-infected, resulting in an estimate of seven (95% CI 2–56) co-infected cases per 100,000 screened women. Among HIV-1 infected women, the prevalence of HCV infection was 7.4% (2/27) (data not shown).

In the univariate analysis, there was an increasing trend of HCV infection among older age-groups (χ^2 ^= 14.6; df = 1; p < 0.01). Also an increasing number of pregnancies were positively associated with HCV infection (Table 1). Higher risk of HCV infection was detected among women older than 39 years (OR_adjusted _= 20.40; 95% CI 4.90–83.33) in the multivariate model. Four or more pregnancies did not remain statistically significant associated when compared to the first pregnancy (OR_adjusted _= 1.64; 95% CI 0.68–3.97) after adjusting for age (Table 2).

**Table 2:** Adjusted association between risk factors and Hepatitis C or HIV-1 infection among pregnant women in Public Health Settings, Central Brazil

Variables	HCV positive OR_adjusted _(95% CI)^a^	HIV-1 positive OR_adjusted _(95% CI)^a^
Age, years		
12–19	1	1
20–29	1.94 (0.66 – 5.75)	3.09 (0.71 – 13.51)
30–39	2.86 (0.84–9.61)	5.38 (1.12 – 25.64)
≥ 40	20.40 (4.90–83.33)	
		
Ethnicity		
White		1
Biracial		1.45 (0.49 – 4.32)
Black		4.90 (1.42 – 16.95)
		
Number of pregnancies		
First	1	
2–3	1.60 (0.72 – 3.52)	
≥ 4	1.64 (0.68 – 3.97)	

^a ^Adjusted odds ratio and 95% confidence interval = OR_adjusted _(95% CI)

In univariate analysis, HIV-1 infection was associated with increasing age up to 39 years old and with women self-referred as black. Other maternal characteristics were not related to HIV-1 infection. In the multivariate analysis, black women had an approximately four-fold greater risk of being infected compared to white subjects, independent of age (OR_adjusted _= 4.90; 95% CI 1.42–16.95). Higher risk of HIV-1 infection was also detected among women older than 29 years (OR_adjusted _= 5.38; 95% CI 1.12–25.64) in multivariate analysis (Table 2).

The outcomes of the HIV-1 screening and the Number Needed to Screen (NNS) were calculated for 0.09% HIV-1 prevalence (Table 3). Numbers needed to screen to prevent 1 case of mother to child HIV transmission was estimated as 4,542 (95% CI; 4,141–5,346) if the combination of antiviral therapy and C-section were 90% and 50%, respectively. NNS was 11,800 (95% CI 10,730–13,928) if the combination of antiviral therapy and C-section were 60% and 37%, respectively. These were the wider range of possibilities for the parameters.

**Table 3:** Outcomes of screening for HIV infection in 10,000 pregnant women

Variables	Prevalence 0.09%
Women screened, n	10 000
Women identified as HIV-1 positive	9
Women receiving test results	8.1 (91%)^a^
Women receiving antiretroviral therapy (ARV)	4.9 – 7.4 (60% – 90%)^a^
Women submitted to elective cesarean	3.0 – 4.1 (37% – 50%)^a^
Cases of mother-to-child transmission (MCT) expected among women without intervention	1.14 – 2.04 (14% – 25%)^a^
Number of cases of mother-to-child transmission prevented with ARV	0.59 (95% CI 0.49–0.64)^b^ 1.59 (95% CI 1.32–1.71)^b^
Number needed to screen (NNS) to prevent one case of infant HIV infection	4,542 (95% CI 4,141–5,346)^c^ 11,800 (95% CI 10,730–13,928)^d^

MCT = Mother-to-Child Transmission

ARV = Antiretroviral Therapy

^a ^values in parenthesis are from literature sources

^b ^values obtained from the model

^c ^NNS calculated from the combination of preventive measures: ARV (90% and cesarean section (50%)

^d ^NNS calculated from the combination of preventive measures: ARV (60% and cesarean section (37%)

## Discussion

In the present study, among 28,561 pregnant women who attended a prenatal screening program, the prevalence of 0.15% for HCV infection was similar to the prevalence of 0.09% for HIV-1 infection. The first prenatal screening was mainly performed by the second gestational trimester among young pregnant women. Increasing age was a risk factor for both HCV and HIV-1 infection, and women self-referred as black were at greater risk for HIV-1 infection. This ethnic pattern is in agreement with the recent epidemiology of HIV-1 infection in pregnant women in Brazil [27].

The overall prevalence of HCV infection among pregnant women of 0.15% in our region is comparable to the figures reported in a similar antenatal program implemented in the neighbouring State in the Centralwest of Brazil [28]. Screening of pregnant women conducted in other Brazilian regions indicated higher HCV frequencies, varying from 1.0% to 2.6% according to the characteristics and risk factors of the population [29-31]. In general, the prevalence of HCV infection is lower among pregnant women compared to the general population [11,12] as shown in our results, since women of childbearing age are usually less than 40 years old. Older age is a known risk factor for HCV infection due to the long period of viral exposure during lifetime. In our study among pregnant women, HCV infection peaked after 40 years of age, which was similar to the most affected age-group reported in a population survey in the US [32].

Around 70% of the suspected cases were confirmed by HCV RNA by PCR, a marker of active viral replication [33] that is compatible with previous reports [11,34,35]. In our study, 15 pregnant women (95% CI 8–25) of 10,000 screened had detectable viremia. This value represents 6 to 13 acquired HCV infections in childhood per year based on the prevalence obtained among the estimated 90 thousand pregnant women in this State (official data) and the rates of mother-to-child transmission of 5% to 8% [36,37]. Genotype 1a (70%) was predominant in our study and is the most frequent circulating genotype in Brazil [38,39].

Routine antenatal HCV screening, although implemented in this Public Health Screening Program for pregnant women (Goiás State), is not mandatory in Brazil, a policy that is in agreement with the current international guidelines [5,40]. In the general population, the screening for HCV is restricted to high risk groups and is not recommended for asymptomatic adults who are not at increased risk [40]. However, if the screening was restricted to high risk group pregnant women, only 40% of those with HCV infection would be identified [41,42]. In Japan, since 2003, HCV screening for the general population and high risk groups, targeting those over 40 years old, was implemented and considered to be cost-effective in comparison to a non-screening strategy [43]. The results of our study indicates that HCV is associated with older age and not associated with ethnicity or increasing number of pregnancies. In this context, the cost-effectiveness of HCV screening in older (>40) populations appears to be an area that presents important opportunities for future investigation. Currently, no intervention can be offered to HCV-infected pregnant women to reduce mother-to-child-transmission [5]. Therefore, HCV antenatal screening does not appear to have benefits that outweigh the risk which contrasts with the screening policy for HIV-1 in pregnant women [44].

The prevalence of HIV-1 infection detected among pregnant women in our setting (0.09%) was similar to the prevalence of HIV-1 infection among women in some developed European and American countries [45]. In Brazil, surveillance data from public hospitals showed around 0.4% HIV-1 prevalence among a representative sample of labouring women between 15 to 40 years old, with regional disparity [46]. We estimated that 4,141 to 13,928 pregnant women need to be screened to prevent one case of HIV vertical transmission (NNS). We estimated that this screening program could enable the prevention of five to fourteen HIV mother-to child transmissions per year if the well-established intervention measures were applied, pointing out the importance of early screening during the gestational period. These figures reflect the HIV- 1 prevalence rate in our region, and it has been found that, in areas with ~50% prevalence such as Sub-Saharan Africa, NNS is 113 to 304 pregnant women [15].

In the 1990s, the HIV-1 vertical transmission rate was estimated as 16% in the southeast of Brazil [47], with a reduction to 7.1% reported in a multicentre study by the year 2001 [22]. Despite the progress in the implementation of screening and free prophylactic measures in recent years in Brazil [20,48], it was estimated that approximately 60% of pregnant women had effective coverage for HIV-1 screening and ~35% of HIV/AIDS positive pregnant women had access to antiretroviral therapy (ARV), with differences among regions [46]. In our setting, the strategy adopted to sign for testing (opt-in) in the antenatal screening program was well-accepted, with minimal refusal rates (0.05%). Besides, the use of dried blood spots on filter paper, as performed by this program, enhances the coverage of the screening by the simplicity of the blood collection/storage and mailing procedure, reducing costs and improving the feasibility of this program [49]. Other large scale screening for infectious disease among neonates and pregnant women have also used dried blood spots [28,49], which have been validated with good correlation with conventional serological tests [50]. In our study, the initial HIV-1 screening was conducted among 60% of women before 14 weeks of gestation, 95% of the testing was performed before 27 weeks, and the final diagnosis was returned within two weeks, timely for the implementation of preventive measures.

Our analysis pointed out that women who self-referred as black had almost 5 fold increase in HIV infection compared to white women in agreement with the Brazilian official surveillance data [20]. This finding is also in concordance with recent analysis from US population showing that black women had nearly 18 times higher prevalence than white women based on CDC estimates in the year 2006 [51]. But we cannot exclude that race/ethnicity may be a surrogate marker of socio-economic condition and differential access to health services as suggested by other studies [46,52]. Other potential risk factors for HIV infection such as early onset of sexual activity, high number of sex partners and having sex with older men were reported among pregnant women [53] and in the general population [20]. One limitation of our study was the inability to assemble data on known risk factors related to sexual behaviour and drug abuse. This program is intended to cover a large population, and it would be unfeasible to collect information about sensitive issues like these in public health settings [54].

The prevalence of HIV-HCV co-infection among pregnant women was low (7 per 100,000); however, among the HIV-1 infected subgroup, 7% were HCV-infected. This frequency of prevalence of HCV infection among HIV-infected individuals is similar to the value reported by the HIV Testing Center regionally [55] and in line with the current recommendations to screen HIV/AIDS patients for HCV virus due to the viruses' common route of transmission and their clinical and therapeutic interactions [56,57].

## Conclusion

The prevalence of HIV and HCV infections were low among pregnant women, with high acceptability rates in the opt-in strategy in primary care. Older maternal age was a risk factor for HCV and antenatal HCV testing does not fulfill the requirements for screening recommendation. The finding of higher risk of HIV-1 infection among black women despite being in consonance with the HIV-1 ethnic pattern in some American regions cannot be ruled out to be a surrogate marker of socio-economic condition. This ongoing screening program is valuable as a surveillance system to monitor HIV-1 and HCV infection patterns for pregnant women and their offspring over an extended period of time.

## Competing interests

The authors declare that they have no competing interests.

## Authors' contributions

ZBC, GCM, MDT, CMTM contributed to study concept and design. WVS and CMTM were responsible for the statistical analysis. MMA, CGF, JVMF, ALM, MMAS revised the manuscript for intellectual content. All authors approved the current version of the manuscript.

## Pre-publication history

The pre-publication history for this paper can be accessed here:

http://www.biomedcentral.com/1471-2334/9/116/prepub
